# Sex in protists: A new perspective on the reproduction mechanisms of trypanosomatids

**DOI:** 10.1590/1678-4685-GMB-2022-0065

**Published:** 2022-10-10

**Authors:** Verônica Santana da Silva, Carlos Renato Machado

**Affiliations:** 1 Universidade Federal de Minas Gerais, Departamento de Genética, Ecologia e Evolução, Belo Horizonte, MG, Brazil.; 2 Universidade Federal de Minas Gerais, Departamento de Bioquímica e Imunologia, Belo Horizonte, MG, Brazil.

**Keywords:** Protists, Trypanosoma cruzi, sexual reproduction, meiosis genes

## Abstract

The Protist kingdom individuals are the most ancestral representatives of eukaryotes. They have inhabited Earth since ancient times and are currently found in the most diverse environments presenting a great heterogeneity of life forms. The unicellular and multicellular algae, photosynthetic and heterotrophic organisms, as well as free-living and pathogenic protozoa represents the protist group. The evolution of sex is directly associated with the origin of eukaryotes being protists the earliest protagonists of sexual reproduction on earth. In eukaryotes, the recombination through genetic exchange is a ubiquitous mechanism that can be stimulated by DNA damage. Scientific evidences support the hypothesis that reactive oxygen species (ROS) induced DNA damage can promote sexual recombination in eukaryotes which might have been a decisive factor for the origin of sex. The fact that some recombination enzymes also participate in meiotic sex in modern eukaryotes reinforces the idea that sexual reproduction emerged as consequence of specific mechanisms to cope with mutations and alterations in genetic material. In this review we will discuss about origin of sex and different strategies of evolve sexual reproduction in some protists such that cause human diseases like malaria, toxoplasmosis, sleeping sickness, Chagas disease, and leishmaniasis.

In the social imaginary, the term “sex” is understood as referring almost exclusively to the sexual act itself (copulation). Biologically, however, sex has a broader definition, being considered not simply an act but rather a crucial strategy of nature that has ensured our survival for thousands of years. The origin of the word “sex” can be traced back to the 12th century, rooted in the Latin word *seccare*, which means cut, section, or division, in reference to male and female sexes (Snoek, 1981). The term has different connotations depending on the context: it can be used in the sense of a sexual relationship (between individuals), in the sense of sex types (male/female or positive/negative mating types), or in a biological sense (which can be succinctly described as a form of genetic exchange or recombination between different organisms) (Bernstein et al., 1984). Scientific evidence suggests that meiotic sex arose on Earth at least 1 billion years ago when early ancestors of eukaryotes began to “experiment” with genetic material exchange (Butterfield et al., 1990; Butterfield 2000; Gibson et al., 2018).

## The Beginning of Life

Life on Earth is estimated to have emerged between 3 and 4 billion years ago amid a hostile environment, constantly bombarded by cosmic radiation and intense UV light coming from the sun. It is known that the concentration of oxygen in the Earth’s atmosphere remained low for a long time, beginning to increase only about 2 billion years ago. However, it was only in the past 500 million years that the atmosphere became completely oxygenated, reaching O_2_ concentrations close to current levels of 21%. This period of oxygenated atmosphere coincides exactly with the development of large, complex life forms (Carver, 1981; Berner et al., 2003; Bekker et al., 2004). High oxygen levels in the atmosphere allowed the formation of an ozone layer and the emergence of aerobic life, which triggered the Cambrian explosion, a geological period marked by accelerated speciation and radiation of different species all over the planet (Hessen, 2008).

The ozone layer, in addition to providing an oxygenated environment for primitive organisms to multiply, served as a barrier against UV rays, which carry sufficient energy to modify chemical bonds and thereby alter the structure of biomolecules, potentially causing damage to nucleic acids, proteins, lipids, and carbohydrates (Hideg et al., 2013; Halliwell and Gutteridge 2015). However, despite the protection provided by the ozone layer, the high oxygen concentrations in the atmosphere and the utilization of this element in cellular metabolism exposed primitive cells to novel damage-inducing agents: reactive oxygen species (ROS) such as superoxide anion (O_2_^−^), hydrogen peroxide (H_2_O_2_), and hydroxyl radical (HO^−^). ROS, released as byproducts of aerobic metabolism (Apel and Hirt 2004), constitute the major endogenous cause of DNA damage, leading to oxidation of nitrogenous bases, which, if not repaired, can result in single-strand breaks, double-strand breaks, DNA adducts, and crosslinks (Dizdaroglu and Jaruga 2012). Although ROS have a short half-life, they can initiate chain oxidation reactions that, in the absence of an effective repair system, may culminate in cell death (Hörandl and Speijer 2018). 

The genetic material of living beings contains all necessary information for cell replication, basal metabolism, and species perpetuation; therefore, maintenance of genetic integrity is fundamental to life. Presumably, the first microorganisms to have emerged were selected under highly oxidative conditions, and those that managed to withstand ROS-induced damage and improve their defense mechanisms, either through synthesis of antioxidant pigments or development of DNA repair mechanisms, were able to ascertain their place in history through evolution (Hessen, 2008; Carletti et al., 2014).

Science has not yet been successful in elucidating eukaryogenesis, the process resulting in the emergence of the first eukaryotes, an evolutionary event of extreme importance to the understanding of the diversity of complex life on Earth (Adl *et al.*, 2012). Evolution scientists developed several models to explain eukaryogenesis (López-García and Moreira, 2015). The most accepted theory is symbiogenesis (Margulis, 1996; López-García and Moreira, 2020), whereby a host cell, probably a member of the phylum Lokiarchaeota (Archaea), incorporated an alphaproteobacterium (mitochondrial ancestor) through endosymbiosis, giving rise to what would be the first eukaryotic cell.

The discovery of symbiotic organisms living inside bacteria (Von Dohlen et al., 2001) and the membrane remodeling process (Godde, 2012; Diekmann and Pereira-Leal, 2013) found both in Archea and in Bacteria have been reinforced the symbiotic model. The sequencing of the first Archea genomes and the knowledge of the transcription machinery of these organisms has revealed that many genes involved in information processing are more similar to eukaryotic genes than to bacterial genes (Spang et al., 2015), suggesting a possible approximation between eukaryotes and Archea.

Phylogenetic analyses based on protein sequences support the model that eukaryotes had emerged as a sister group or from the TACK Archea´s superphylum, composed of phyla Thaumarchaeota, Aigarchaeota, Crenarchaeota and Korarchaeota (Guy and Ettema 2011). By comparative genomics it was possible to observe specific signs of eukaryotic proteins (ESP) in organisms of the TACK superphylum of Archea, such as proteins involved in processes of trafficking, cell division, transcription and translation (Hartman and Fedorov 2002; Guy and Ettema 2011; Yutin and Koonin 2012; Williams et al., 2013; Spang et al., 2015).

In recent years, 16S rRNA gene sequences have been identified in Archea that live more than three thousand meters deep in the mid-Arctic ocean range, in the hydrothermal field known as Loki’s castle (between Greenland and Norway) (Spang et al., 2015). After phylogenetic analysis, the genome of Lokiarcheota, a new clade within the TACK superphylum of Archea, was identified and characterized. The Lokiarcheota group according to phylogenetic analyse by conserved proteins, forms a monophyletic group with eukaryotes, being the most ancestral group and considered the gap between prokaryotes and eukaryotes (Spang *et al.*, 2015). Other organisms, from sister groups to Lokiarchetoas, were discovered in estuarine sediments of the White Oak River (USA), being named Thorarcheota. Organisms of this group would be able to degrade organic matter, fix inorganic carbon and reduce sulfuric acid (Seitz et al., 2016), suggesting that some characteristics of basal metabolism of current eukaryotes were already present in primitive prokaryotes.

Through metagenomic studies using gene sequences of a conserved ribosomal protein (RP15), other Archea lineages have also been discovered in recent years, such as the groups Odinoarcheota, found in hydrothermal vents in Yellowstone National Park (USA) and in the Radiata Pool (New Zealand) and the Heimdallarcheota group discovered also at Loki Castle and Aarhus Bay (Denmark). In view of the diversity of the latest Archeas discovered and supported on analysis of protein and rRNA sequences, Zaremba-Niedzwiedzka et al. (2017) grouped the Lokiarcheota, Thorarcheota, Odinoarcheota and Heimdallarcheota, all in the Asgard superphylum, the closest group to complex eukaryotes (Zaremba-Niedzwiedzka *et al.*, 2017), The Asgard group has gene sequences unique to eukaryotes, which encode proteins involved with membrane trafficking, vesicle formation and transport, ubiquitins, and cytoskeleton formation (Eme et al., 2017; Zaremba-Niedzwiedzka *et al.*, 2017; Imachi et al., 2020).

Imachi et al. (2020), managed to isolate and cultivate for the first time in the laboratory a representative of the Asgard group, which they named *Candidatus Prometheoarchaeum syntrophicum* strain MK-D1. These microorganisms were observed under a microscope, drawing attention to the fact they have long “tentacles” intertwined with each other. The researchers were also able to discover that they are able to degrade amino acids present in the medium anaerobically and through a cooperative relationship with other microorganisms. When in the presence of different bacteria, the *Prometheoarchaeum syntrophicum,* they were able to use the available oxygen from the medium in a syntrophic way. The researchers who isolated, cultured and characterized these organisms suggest an order of possible events for the process of eukaryogenesis, which would be: intertwining, engulfing and endogenizing bacteria, known as The Entangle-Engulf-Endogenize (E3) model (Imachi *et al.*, 2020). Thus, drawing the sequence of events that may have enabled the emergence of the first eukaryotic cells on Earth (protoeukaryotes), wich would drive evolutionarily into the present-day eukaryotes.

According to Sagan and Margulis (1987), the kick-off of sexual reproduction was a similar cannibalistic event among unicellular organisms inhabiting primitive Earth. In periods of stress, such as variations in pH, salinity, and nutrient availability, primitive cells might have phagocytized each other, leading to karyotypic combination and possibly gene exchange. Such a case of “poor digestion” might have provided an adaptive advantage to cannibalistic cells over generations through increased genetic variability, thereby promoting the emergence of sexual reproduction (Sagan and Margulis, 1987).

The oldest record of eukaryotic fossils dates back to approximately 1 billion years and is directly related to the evolution of sex (Gibson et al., 2018). The red alga *Bangiomorpha pubescens* n. gen., n. sp., belonging to the group of bangiophytes, was found in the Hunting formation on Somerset Island, Arctic Circle, Canada (Stewart ,1987). It has at least two distinct phases of spore production, comparable to the sexual phases found in modern *Bangia* (Butterfield, 2000). This scientific evidence suggests the existence of sex in eukaryotic cells since ancient times, having protists, the most primitive eukaryotes, as the earliest representatives of sexual reproduction.

## SEX: Origin and Evolution

Dealing with the most varied types of damage to genetic material through the development of different repair mechanisms was a triumphant event in the evolutionary history of living beings, and some authors support the hypothesis that sex emerged as a direct consequence of such mechanisms (Bernstein et al., 1984; Michod and Levin, 1988; Charlesworth, 1989; Long and Michod, 1995; Bernstein *et al.*, 2017). At least two fundamental characteristics should be considered when studying the origins of sex: (i) recombination of genetic material involves the exchange of genetic information between two homologous chromosomes and (ii) participating chromosomes are usually derived from two different individuals (Bernstein *et al*., 1984, 2017). 

In a primitive environment, organisms that were able to recombine their genetic material generated a new set of genes and thus acquired adaptive advantages. For example, it is known that DNA repair induced by radiation damage involves genetic recombination. The fact that some recombination enzymes also participate in meiotic sex in modern eukaryotes further reinforces the idea that sexual reproduction emerged as consequence of specific mechanisms to cope with mutations and alterations in genetic material (Rothschild, 1999; Hörandl and Speijer, 2018).

In 1964, the geneticist Herman Muller hypothesized that, in the absence of recombination, the genome of an asexual population would irreversibly accumulate deleterious mutations (Muller, 1964). This process, which later became known as Muller’s ratchet (Felsenstein and Yokoyama, 1976), is based on the assumption that a population of finite size that reproduces asexually tends to accumulate deleterious mutations over time. The proportion of the population unaffected by mutations would become smaller and smaller and more susceptible to environmental variations, favoring the survival of mutated individuals. This process would be irreversible, given that it is unlikely that any member of the population would reverse back to its wild traits. By contrast, in a sexually reproducing population, recombination between individuals with different mutations could restore original traits, thus allowing the survival of the population. This argument is considered by some authors an explanation to the origins of sex (Michod and Levin, 1988; Rothschild, 1999; Walker et al., 1976), with recombination having emerged as an adaptive strategy.

It is known that mutation rates may increase under stress conditions (Hall, 1992; Foster, 1999; Goho and Bell 2000). Environment-dependent variations in recombination and mutation rates may indicate that genomic processes, such as elimination of DNA-damaging agents, are sensitive to the physiological state of the organism. For instance, individuals frequently exposed to stress may have high rates of DNA double-strand breaks resulting from repeated attempts to survive stressful conditions (Agrawal et al., 2005). This fact may provide insight into processes related to the unstable and unpredictable environment in which primitive cells survived and grew in complexity.

In eukaryotes, there is some evidence that recombination is a ubiquitous mechanism that can be stimulated by DNA damage. Bernstein and Johns (1989) demonstrated the relationship between DNA repair and sexual reproduction in yeasts. Vegetative cells of *Schizosaccharomyces pombe* showed an 8-fold increase in sexual reproduction after exposure to hydrogen peroxide (H_2_O_2_), a compound that induces oxidative DNA damage (Bernstein and Johns, 1989; Rothschild, 1999). Another example of recombinational sex in eukaryotes is seen in the green alga *Volvox carteri*, whose sexual reproduction can be induced by thermal shock (Kirk and Kirk, 1986) and inhibited by antioxidants, indicating that sexual induction in these organisms is mediated by oxidative stress (Nedelcu and Michod, 2003)*.* These findings support the hypothesis that ROS-induced DNA damage can indeed promote sexual recombination in eukaryotes, which might have been a decisive factor for the origin of sex on primitive Earth. 

In a phylogenetic study using elongation factor 1 alpha (EF-1 alpha), a protein involved in the highly conserved translation machinery of eukaryotes, Dacks and Roger (1999) proposed that sex might have been facultative in the common ancestor of eukaryotes. Since then, there have been numerous reports of sexual reproduction in eukaryotic pathogens previously believed to be solely asexual (Malik et al., 2008; Lahr et al., 2011), supporting the idea that meiotic sex may be a basic trait in all eukaryotes. Such a hypothesis is reinforced by genetic studies on the protozoa *Trichomonas vaginalis* and *Giardia intestinalis* (syn. *lamblia*), organisms descended from a common lineage that diverged early in the evolutionary history of eukaryotes. Their common ancestor carried meiosis-specific genes, which, according to some authors, suggests the presence of meiotic genes and sex in primitive eukaryotes (Ramesh et al., 2005; Malik *et al.*, 2008). Evidence of sexuality in other species previously considered asexual, such as individuals of the genus *Leishmania* (Akopyants et al., 2009) and the primordial sexual ancestor of amoebas (Lahr *et al.*, 2011), has pointed to the existence of cryptic sex in different microorganisms (Heitman, 2010; Ramírez et al., 2012).

Organisms can be classified as obligate sexual (i.e., reproduction occurs exclusively through meiosis), parasexual (i.e., non-meiotic recombination with ploidy reduction, found in some unicellular eukaryotes) (Pontecorvo et al., 1953; Mishra et al., 2021), obligate asexual, or facultative sexual (i.e., sexual and asexual reproduction are present). Facultative sex is found in various organisms (Dacks and Roger, 1999; Otto, 2009), from plants that reproduce by cross-pollination, self-pollination, and vegetative reproduction (Holsinger, 2000) to invertebrates (Suomalainen, 1962) that rely on both sexual reproduction and parthenogenesis, such as the Cape honey bee (*Apis mellifera capensis*). Of note, there have been surprising reports of parthenogenesis in several vertebrates, including snakes, lizards, birds, and sharks (Booth et al., 2012). Unicellular eukaryotes reproduce mostly asexually but may use sexual reproduction occasionally (Tibayrenc et al., 1991), which legitimizes the presence of meiotic genes in these organisms. Facultative sexual organisms have the ability to switch between sexual and asexual reproduction depending on individual and environmental conditions (Ram and Hadany, 2016). This observation reinforces the hypothesis that sex originated through genetic recombination in response to adverse conditions in the primitive environment.

## Emergence of gametes

According to Butterfield (2000), the morphological differentiation ability, multicellularity, and size of eukaryotes allowed them to prevail over prokaryotes on a planet monopolized by perfectly adapted prokaryotic life forms in the absence of a mass extinction event. During this period, sex was critical to eukaryotic evolution, as it introduced a significant evolutionary advantage by enhancing morphological variability (Butterfield, 2000).

The current abundance of unicellular eukaryotic clades does not suggest that multicellular complexity was the driving force of sexual evolution (see Bell, 1982); rather, it lends support for theories proposing that the emergence of recombinational sex contributed to the appearance of multicellular life. Organisms that were able to recombine their genetic material might have acquired differentiated physiological and morphological traits over time, culminating in cell specialization and increased complexity. Asymmetric cell division, for instance, would have produced different characteristics in sister cells, leading to specialization and intraorganizational division of labor (Horvitz and Herskowitz 1992; Szathmáry and Smith 1995; Kirk, 1998). This scenario was likely the origin of multicellular eukaryotes and their specialized reproductive cells (gametes). As discussed by Kondrashov (1997), multicellularity might have been the result of a replacement of somatic mitosis by reproductive mitosis; the latter process would afford a multicellular mass of identical cells, which, upon exposure to different microenvironments, could have differentiated into specific cell lines. 

Sexual reproduction requires the fusion of distinct gametes. Most unicellular eukaryotes are isogamous, having gametes of similar size and mobility but different mating types (Fraser et al., 2004; Ahmed et al., 2014; Branco et al., 2017; Branco *et al.*, 2018). Isogamy can be found in organisms such as amoebas (e.g., *Dictyostelium discoideum*), fungi (e.g., *Saccharomyces cerevisiae*), trypanosomatids (e.g., *Trypanosoma brucei*), dinoflagellates (e.g., *Polykrikos kofoidii*), and algae (e.g., *Ascoseira mirabilis* and *Carteria palmata*) (Lehtonen et al., 2016). These organisms have morphologically identical gametes that mate disassortatively (without preferences), though mating is scarcely ever seen between equal mating types (Hoekstra, 1987). Sexual reproduction is asymmetrical, and reproductive cells exhibit genetic, physiological, and behavioral differences despite having high levels of morphological similarity. Only cells of different mating types can merge and reproduce sexually, promoting genetic variability. The current existence of sexual asymmetry in unicellular organisms may provide explanations for the evolution of gamete fusion in primordial eukaryotes (Hadjivasiliou and Pomiankowski, 2016; Hadjivasiliou and Pomiankowski, 2019).

Hadjivasiliou and Pomiankowski (2016) proposed a hypothesis based on the strength of pairwise interactions between different gamete types. According to their model, novel mating types only spread if they are able to interact strongly with existing mating types, and the strength of pairwise interactions between existing types limits the attraction and recognition of new variants. However, it is possible for multiple mating types to evolve if specialization does not restrict gamete interactions. This interaction model also explains why, in species with multiple mating types, not all types exist at the same frequency (Douglas et al., 2016).

In recent decades, several hypotheses have been developed in an attempt to clarify the evolution of isogamous mating types (Billiard et al., 2011, 2012; Perrin, 2012). One such hypothesis predicts that the emergence of different types contributes to preventing mating between genetically related individuals, minimizing, for instance, the deleterious consequences of inbreeding (Charlesworth and Charlesworth, 1979; Uyenoyama, 1988; Czárán and Hoekstra, 2004). 

According to Hadjivasiliou and Pomiankowski (2019), the fact that sex cells from the same mating type cannot reproduce with each other restricts the choice of mating partners and hinders reproduction within the population. The authors also noted, however, that different mating types are present in the sexual reproduction of all eukaryotes, from invertebrates to vertebrates. This observation motivated the authors to develop mathematical models to explain the evolution of pairwise reproduction strategies. A possible explanation lies in the occurrence of better recognition and communication between different mating types than between equal types, given that communication between cells is mostly mediated by surface ligands and protein receptors. Using mathematical modeling, the authors showed that natural selection tends to favor asymmetric signaling, as exemplified by the interaction of receptor A with ligand B or receptor B with ligand A. Asymmetric mutants would be favored by avoiding the production of ligands that could clog or activate their own surface receptors; thus, as a result, different types of cells would recognize each other more easily and mate more efficiently (Hadjivasiliou and Pomiankowski 2019). This model offers a possible reconstruction of the evolutionary steps in the rise of sexual organisms from the first protoeukaryotes as well as of the origins of specialized sex cells such as gametes.

The emergence of sexual reproduction (syngamy, genetic recombination, and meiosis) is a milestone in the evolutionary history of complex living beings, as it allowed greater variability, Schopf et al. (1973), provided the ability to remove deleterious mutations (Muller, 1964), and stimulated the development of new species (Stanely, 1975). During meiosis, sexual organisms complete a ploidy cycle, undergoing a diploid phase and a haploid phase. And, although asexual organisms do not experience ploidy changes, they can also exhibit a ploidy cycle, characterized by alternation between duplication and reduction of genetic content depending on environmental conditions. Ploidy cycling decreases the mutation load of cells compared with permanent diploidy or polyploidy. The ploidy cycle found in ancestral asexual organisms may have promoted the origin of sex by providing pre-existing and regular mechanisms of gene reduction immediately after syngamy (Kondrashov, 1994). It is postulated that, during this evolutionary process, chromosomal rearrangement and recombination through fusion of haploid gametes and reduction of diploidy via meiosis gave rise to sexual reproduction in the last eukaryotic common ancestor, as these mechanisms are ubiquitous in all complex eukaryotes, given the expression of meiosis-related genes (Goodenough and Heitman, 2014).

## Meiosis

Meiosis is the major source of genetic variability in eukaryotic individuals. This process has been responsible for the formation of gametes throughout evolution and the maintenance of ploidy in sexually reproducing organisms. In most species, the first meiotic (reductional) division involves separation of homologous chromosomes and the second meiotic (equational) division results in separation of sister chromatids, as occurs in mitosis. For meiotic reduction division to be successful, it is necessary, first and foremost, the formation of the synaptonemal complex, responsible for the correct pairing of homologous parental chromosomes, forming bivalents. Once paired, two non-sister chromatids from homologous chromosomes can undergo a process known as crossing over (Grelon, 2016), creating connection structures called chiasmata (Kleckner, 2006; Neale and Keeney, 2006). These physical connections between parental chromosomes are seen during meiotic prophase I (meiotic recombination) (Page and Hawley, 2003). The occurrence of at least one crossing over per bivalent allows adequate segregation of homologous chromosomes and promotes gamete viability. Crossing overs lead to allelic recombination within chromosomes and shuffle parental chromosomes in daughter cells, being a relevant contributor to genetic variability over generations (Alberts et al., 2010; Grelon, 2016). Finally, meiotic reduction division compensates for the chromosomal duplication that occurs during gamete fusion (Alberts *et al.*, 2010).

It is known that meiosis-specific genes are well conserved in most eukaryotes. Phylogenetic analysis identified 34 genes encoding proteins participating in the recombination machinery of cells, cohesion between sister chromatids, and synaptonemal complexes in several eukaryotes; 12 of these genes were found to be involved exclusively in meiosis, namely *SPO11-1*, *SPO11-2*, *HOP1*, *HOP2*, *MND1*, *DMC1*, *MSH4*, *MSH5*, *MER3*, *ZIP1*, *ZIP4*, and *REC8* (Malik et al., 2007; Malik *et al.*, 2008)

Recently, a possible sexual ancestor of eukaryotes was identified by phylogenetic analysis of meiosis-specific proteins (Hofstatter and Lahr 2019). In agreement with the observations of Grelon (2016), Hofstatter and Lahr (2019) defended the hypothesis that the meiotic machinery evolved from the DNA repair machinery of a common ancestor, in this case an Archaea, through duplication of ancestral genes. According to the authors, it is no wonder that proteins related to sexual processes are widely distributed in eukaryotes, and there are no differences in the distribution patterns of meiotic proteins between sexual eukaryotes and those believed to be asexual. In discussing the origin and evolution of sex, the authors pointed out that some members of the protist kingdom carry many, but not all, meiosis genes. The most parsimonious hypothesis to explain this evolutionary pattern suggests the occurrence of gene loss events throughout evolution (Ramesh et al., 2017). Taken together, these findings indicate that the last eukaryotic common ancestor already had the necessary machinery for meiosis, being therefore able to perform sexual reproduction. If sex had evolved separately on more than one occasion along the evolutionary trajectory of living beings, meiotic sex would likely exhibit different mechanisms of action, which does not hold true, for meiosis is a well-conserved mechanism shared among all eukaryotic lineages (Hofstatter and Lahr, 2019; Hofstatter *et al*., 2020).

## SEX in some pathogenic protists

Sex in the sense of cell fusion, nuclear fusion, and meiosis only occurs in eukaryotes and is closely related to the exchange and recombination of genetic material between individuals. Given that the evolution of sex is associated with the origin of eukaryotes (Cavalier-Smith, 2002; Cavalier-Smith, 2010; Weedall and Hall, 2014) and protists are the most ancestral representatives of the group, it is not surprising that many extant protozoa are capable of sexual reproduction. 

The kingdom of protists (Protozoa) has the greatest heterogeneity, including unicellular and multicellular algae, photosynthetic and heterotrophic organisms, as well as free-living and pathogenic protozoa. Protist individuals can be found in almost all taxa of the classification of eukaryotes (Burki et al., 2020) and are known to have inhabited Earth since ancient times and are currently found in the most diverse environments, from a simple pool of water to diseases infecting millions of people worldwide, as is the case of trypanosomatids that cause sleeping sickness, Chagas disease, and leishmaniasis. 

The notion that there are far more protist species than those currently described is widely accepted. Some protist exemplars can survive under extreme environmental conditions that would be expected to kill all living beings, such as the red alga *Cyanidium caldarium*, found in acidic environments with pH below 1 (Rothschild and Mancinelli, 2001). Because of their heterogeneity, protists have been used as model organisms for studies on the most varied biological processes, aiding in the understanding of conserved and divergent evolutionary processes (Collier and Rest, 2019).

Research on processes that allowed protists to modify and perpetuate their existence throughout history can provide a broader and extremely enriching scientific perspective. Understanding how these ubiquitous organisms conquered their place on planet Earth opens new questions in the most diverse areas, including reproduction biology. The fact that reproduction strategies of parasitic protists, particularly pathogenic ones, have been the subject of intense research and debate (Tibayrenc et al., 1990; Tibayrenc and Ayala, 2002; Weedall and Hall, 2014) reflects how much we still have to learn about these organisms.

## Examples of sexual reproduction in protists

As unicellularity and sexual differentiation are not readily apparent in a large number of protists, it was long believed that these organisms were only capable of vegetative (asexual) reproduction. Currently, it is known that protozoa such as *Plasmodium*, *Babesia*, *Theileria*, *Toxoplasma*, *Eimeria*, *Cryptosporidium*, *Trypanosoma*, *Leishmania*, *Giardia*, *Trichomonas*, and *Entamoeba* have a sexual phase in their life cycle (Weedall and Hall, 2014).

According to Weedall and Hall (2014), when discussing sex in parasitic protists, it is important to take into account some characteristics regarding the organism’s classification both as “protist” and as “parasite.” The simple fact that protists are single-celled organisms indicates that they differ considerably from multicellular eukaryotes. As for reproduction, both mitotic and meiotic divisions can be reproductive strategies, because they generate new cells. Therefore, it is perfectly possible for reproduction in protists to happen solely by mitosis (clonal or asexual reproduction). In multicellular eukaryotes, diploid cells divide by mitosis and gametes are haploid, whereas the reproduction pattern and lifestyle of protists are completely different. Some obligate sexual protists, such as *Plasmodium*, spend a great part of their life cycle as haploid cells, entering a diploid phase shortly after zygote formation, that is, before meiosis (Sinden and Hartley, 1985; Sinden *et al*., 1985). Thus, meiosis may not be a necessary process for some species that rely on clonal reproduction, but, in other species, haploid forms may be an essential stage. The second point that should be considered is that parasitism is an interspecific relationship that arose independently in different species over time. It is important to remember that just as genetically distant parasites coexisting in similar niches may exhibit similar adaptations, evolutionarily close organisms may have completely different life cycles (Weedall and Hall, 2014), which can be observed in a multitude of species in the protist kingdom. 

Among sexually reproducing parasites, some are heterogamous, characterized by having notoriously distinct male and female gametes, and some are isogamous, showing no morphological differences between gametes. For species in which sexual reproduction is not evident, different methods can be used to prove or infer the existence of sexuality. The most direct approach is to visualize the presence of gametes and fusion events *in vitro* or *in vivo*. Another strategy is to analyze genetic variations that could indicate sexual reproduction in natural populations. A third method is the identification of meiosis genes, which can evidence a possible mean for sexual reproduction, as already reported in several organisms (Malik et al., 2008; Ehrenkaufer et al., 2013; Hofstatter et al., 2018, 2020). In the next sections, we will address two examples of pathogenic protists that reproduce sexually using different gametes that can be visualized by microscopic techniques: *Plasmodium* spp. and *Toxoplasma gondii.*

## Sexual reproduction in *Plasmodium* spp.

Malaria is a disease caused by *Plasmodium* spp., unicellular eukaryotes belonging to the phylum Apicomplexa. Different species of *Plasmodium* infect different organisms, from invertebrates to vertebrates. *Plasmodium* species that can infect humans include *P. falciparum*, *P. vivax*, *P. malariae*, *P. knowlesi*, and *P. ovale* (divided in two subspecies, *P. ovale curtisi*and*P. ovale wallikeri*) (Sutherland et al., 2010). *P. falciparum* is responsible for most deaths in humans. Malaria is more prevalent in tropical and subtropical regions. According to WHO estimates (2019), in 2018, 93% of global cases of malaria were recorded in Africa (Su *et al.*, 2020). 

The malaria parasite goes through different phases during its life cycle. Sporozoite forms are inoculated in vertebrate hosts as infected *Anopheles* mosquitoes feed on blood. After successive mitotic divisions, parasites differentiate into merozoites in the liver and into schizonts and trophozoites in red blood cells (Tavares et al., 2013). For reproduction, *Plasmodium* undergoes gametocytogenesis in the vertebrate host, whereby some parasites, depending on environmental conditions (e.g., metabolite concentrations in host tissues), enter a sexual stage and originate male (microgametocytes) and female (macrogametocytes) gametocytes. 

Mature gametocytes circulate in the blood of the vertebrate host until they are ingested by female *Anopheles* mosquitoes. Once they reach the midgut of the invertebrate host and are exposed to the necessary conditions for maturation (low temperature and high pH in the presence of xanthurenic acid), gametocytes differentiate into male and female gametes (Billker et al., 1997, 1998, 2004). During maturation, each microgametocyte undergoes a process known as exflagellation, originating eight male gametes through successive mitotic divisions. By contrast, each macrogametocyte maturates without division and forms one female gamete. Gamete fusion results in the formation of a diploid zygote, which differentiates first into an ookinete and then into an oocyst containing thousands of haploid sporozoites. After maturation, these sporozoites migrate to the mosquito’s salivary glands, from where they are reintroduced into a vertebrate host during blood sucking (Beri et al., 2018; Su et al., 2020). Thus, *Plasmodium* spp. spend most of their life cycle as haploid forms, exhibiting diploidy only during the zygote and ookinete stages, when meiosis and genetic recombination occur (Sinden and Hartley, 1985; Sinden *et al.*, 1985; Sinden, 1991, 2009). Therefore, it can be said that the malaria parasite has two reproductive stages, an asexual stage dependent on successive mitotic divisions that occur both in vertebrate and invertebrate hosts and a sexual reproduction phase with gamete formation, fusion, and meiosis, taking place in the invertebrate host only.

## Sexual reproduction in *Toxoplasma* gondii

Considered one of the most common infectious diseases in the world, toxoplasmosis affects about one-third of the world’s population (Weiss and Dubey, 2009). It is caused by the microscopic obligate intracellular eukaryote *T. gondii*. This parasite was first described in 1908, found in rodents in Africa (Nicolle and Manceaux 1908, 1909) and in rabbits in Brazil (Splendore, 1908). *T. gondii* is known to infect several warm-blooded animals, including humans. The first reported case was of a child with signs of meningoencephalitis in 1923 in Prague, Czech Republic (Janku, 1923; Wolf and Cowen, 1937; Wolf *et al.*, 1939). *T. gondii* infection may occur via ingestion of meat, food, or water contaminated with cysts or oocysts, as well as by blood transfusion and vertical transmission. Congenital infection can lead to abortion or neurological malformation in infants, having been described for the first time in a newborn in 1938 (Wolf *et al.*, 1939; Dubey *et al.*, 2012). 

The parasite undergoes three basic developmental stages during its life cycle: sporozoite, tachyzoite, and bradyzoite Dubey (1998). Humans, small rodents, and other vertebrates are intermediate hosts of *T. gondii*, whereas felines are definitive hosts, given that sexual reproduction occurs in the intestine of this group of animals. Such host specificity can be attributed to biochemical characteristics inherent to the feline intestine. Cats are the only mammals that do not metabolize linoleic acid, causing an increase in the levels of this acid in the intestinal microenvironment, which contributes to the development of sexual stages of *T. gondii* (Di Genova et al., 2019).

When cats feed on animals contaminated with *T. gondii* cysts, bradyzoites are released from cysts into the feline intestine and invade intestinal epithelial cells. Once inside cells, the parasites undergo mitotic division (schizogony), giving rise to merozoites, which develop into male (microgametes) or female (macrogametes) gametes.

Gamete formation in felines begins only two days after ingestion of tissue cysts Dubey (1998). Merozoites undergo five asexual stages (A to E) in intestinal cells (Dubey and Frenkel, 1972). The last two stages, type D and E meronts, are fundamental for gamete differentiation and formation. At the end of meront development, gametocyte precursor cells, known as macrogamonts and microgamonts, are formed, subsequently giving rise to female and male gametes, respectively. One microgamont produces a mean of 12 microgametes, whereas one macrogamont produces one macrogamete only (Tomasina and Francia, 2020). After gamete fertilization within intestinal cells, thousands of immature diploid oocysts are formed and eliminated in the feces of felines. In up to five days after elimination, oocysts undergo sporulation and are further divided by meiosis, forming haploid sporozoites, which remain inside oocysts indefinitely. Only after sporulation do oocysts become mature and infectious, being able to contaminate water and food ingested by any warm-blooded animal, including humans (Hill and Dubey, 2002; Weiss and Dubey, 2009; Halonen and Weiss, 2013). 

For *T. gondii*, it was observed that zygote formation was not much effective when hosts were infected by a single parasite strain, suggesting that, in this protozoan, sexual reproduction is only advantageous when hosts are infected simultaneously with different strains, thereby increasing the possibility of genetic diversity (Ferguson, 2002). 

Although *P. falciparum* and *T. gondii* belong to the same phylum (Apicomplexa), the reproductive strategies of these parasites, adopted for over thousands of years, are completely different. Both have asexual and sexual reproduction phases with gamete formation; however, interaction with a great variety of hosts has shaped their reproduction. *P. falciparum* can infect both vertebrates and invertebrates, but sex (gamete fusion) only occurs in invertebrate hosts. By contrast, *T. gondii* has only been detected in endothermic vertebrates and reproduce sexually (with gamete formation) in a specific group of vertebrates, the felines. The meiotic process of *T. gondii* occurs outside the host. Such examples demonstrate how the evolution of well-conserved processes and mechanisms such as sex can proceed along several paths.

## SEX in trypanosomatids

Whereas for some protozoa the sexual reproduction phase with gamete formation is well described, as in the examples cited above, for others, such as trypanosomatids, sexual reproduction is not so evident, necessitating detailed research. Trypanosomatids are a group of flagellate parasites of the order Kinetoplastida, belonging to Euglenozoa group, (Maslov et al., 2001; Hampl et al., 2009; Burki et al., 2020). During their life cycle, these organisms have high phenotypic plasticity, hindering studies on their reproductive forms. Furthermore, there are methodological limitations to investigating the sexual reproduction of trypanosomatids, because, as highlighted by Gibson and Peacock (2019), the techniques we need to “see” these subjects have not yet been created. 

In recent decades, trypanosomatids that cause globally known diseases such as leishmaniasis, African trypanosomiasis (sleeping sickness), and American trypanosomiasis (Chagas disease) were found to be capable of carrying out meiotic events and genetic exchange. These discoveries were mainly provided by the advancement of analytical techniques, including fluorescent proteins (Gibson and Peacock, 2019) and whole-genome sequencing (Rogers et al., 2014; Inbar et al., 2019; Schwabl et al., 2019). 

## Sexual reproduction in *Leishmania* spp.

Leishmaniasis, a disease caused by *Leishmania* spp., is transmitted by the bite of phlebotomine females (sandflies) on vertebrate hosts, such as dogs, rodents, marsupials, and humans. The disease is prevalent in the tropics, subtropics, and southern Europe, occurring in more than 98 countries, with about 12 million cases worldwide. Leishmaniasis can be cutaneous, causing skin wounds, or visceral (also known as *kala-azar*), causing damage to various internal organs (e.g., spleen, liver, and bone marrow). Reports of the disease have been made since 2500 BCE, as identified in ancient writings and molecular archaeological finds (Alvar et al., 2012; Akhoundi et al., 2016). 

The life cycle of *Leishmania* spp. is divided into three main phases: amastigote, procyclic promastigote, and metacyclic promastigote. When a phlebotomine (vector) feeds on the blood of an infected host, it ingests amastigote forms of the parasite. Upon reaching the stomach of the insect, amastigotes develop into procyclic promastigotes, which later migrate to epithelial cells of the digestive tract, where they undergo binary fission. Then, the parasites migrate to the anterior portion of the intestine, where metacyclogenesis occurs, resulting in differentiation into infectious forms called metacyclic promastigotes. Infectious forms are eliminated by the insect during blood feeding, infecting the vertebrate host. Within the host, metacyclic promastigotes can invade various types of cells, namely fibroblasts, dendritic cells, neutrophils, and macrophages, through phagocytosis. Inside cells and protected by a parasitophorous vacuole, the parasite differentiates into amastigotes, which undergo successive divisions. Once the infected cell is ruptured, amastigotes are released into the bloodstream and may invade new blood cells until an insect feeds on the host’s blood, restarting the cycle. 

Despite the non-observance of gametes in *Leishmania*, the existence of naturally occurring hybrids and presence of meiosis orthologs in the genome of these parasites indicate the possibility of sexual reproduction (Heitman, 2006; Heitman, 2010). The first evidence of genetic exchange events in *Leishmania* was reported in the last decade (Akopyants et al., 2009). In the referred study, phlebotomine flies co-infected with parental strains carrying different selection markers produced a hybrid progeny with both markers. Simple nucleotide polymorphism experiments confirmed that the analyzed progeny was heterozygous, unlike their homozygous parents. DNA analysis revealed that the parents were diploid, as were most hybrids. However, about 38% of the hybrids were triploids, suggesting fusion between diploid cells (without meiotic reduction) and haploid gametes. Another hypothesis raised was the occurrence of parasexuality, as observed in the fungus *Candida albicans*. *Leishmania* spp. could undergo a diploid-tetraploid-diploid/parasexual cycle, in which triploid organisms would be the intermediates. Genetic exchange events in *Leishmania* were also observed in experiments with parasites carrying fluorescent reporter genes, allowing identification of hybrids by fluorescence microscopy (Calvo-Álvarez et al., 2014). Although no gamete form of *Leishmania* has yet been observed, to date, studies have suggested that genetic exchange events from fusion between cells may occur in *Leishmania* spp. and that sex might be cryptic (Akopyants *et al.*, 2009; Heitman, 2010; Rogers et al., 2014; Sterkers et al., 2014). Such findings may instigate researchers around the world to unravel the evolution and mechanisms of sex in *Leishmania* spp.

## Sexual reproduction in *Trypanosoma brucei*

*T. brucei*, the causative agent of human and animal African trypanosomiasis (Isaac et al., 2017), is a trypanosomatid known to reproduce sexually (Peacock et al., 2014). The parasite has two types of hosts, the tsetse fly (*Glossina*), as the invertebrate host, and several mammals, as vertebrate hosts. Human African trypanosomiasis is endemic to Africa, given that its vector, the tsetse fly, only occurs in that continent. There are three subspecies of the parasite, *T. brucei brucei*, *T. brucei gambiense*, and *T. brucei rhodesiense*. *T. brucei brucei* is responsible for animal African trypanosomiasis, popularly known as nagana, which affects cattle, pigs, camels, sheep, and other animals. The main causative agents of human trypanosomiasis are *T. brucei gambiense*, responsible for more than 90% of cases in Africa (Simarro et al., 2010), and *T. brucei rhodesiense*, which can cause death if the host does not receive early diagnosis and treatment (Brun et al., 2010). 

*T*. *brucei* has a complex life cycle, encompassing several phases: metacyclic trypomastigote, blood trypomastigote, procyclic trypomastigote, and epimastigote. Tsetse flies, when feeding on blood from hosts contaminated with *T. brucei*, ingest blood trypomastigotes, which differentiate into the replicative form, procyclic trypomastigote, in the fly midgut. Leaving the intestine, parasites differentiate into epimastigotes and migrate to salivary glands, where they differentiate into the infectious form, metacyclic trypomastigote. Upon entering vertebrate hosts, such as humans, infectious forms differentiate into replicative forms (blood trypomastigotes), which can infect various parts of the body or remain in circulation until being ingested by flies during blood feeding (Vickerman, 1985).

Hybridization events in *T. brucei* were first described in the late 1980s, when, after infecting an invertebrate host with two different strains of the parasite, researchers were able to isolate hybrid cells during the trypanosome transmission cycle (Jenni et al., 1986). Evidence of the formation of *T. brucei* hybrids by nuclear fusion in tsetse flies was reported soon after (Paindavoine et al., 1986; Wells et al., 1987). However, only two decades later was it possible to observe the location of hybrid *T. brucei* cells, that is, in the salivary glands of the tsetse fly (Gibson et al., 2008). *In vitro* studies were only able to identify the meiotic phase of the parasite (Peacock et al., 2014). Analysis of DNA content throughout the *T. brucei* life cycle revealed haploidy in tsetse fly salivary glands and an increase in expression of meiosis genes moments before cell fusion (Peacock *et al*., 2011, 2014). Cells identified as gametes of *T. brucei* are haploid, morphologically distinct from parental cells, exhibit a certain interaction *in vitro*, and have two possible conformations regarding the presence of nuclear (N) and mitochondrial (K) DNA. 2K1N cells have one nuclear DNA and two mitochondrial DNAs, and 1K1N cells have one nuclear DNA and one mitochondrial DNA (Peacock *et al.*, 2014). In a recent study, some intermediate stages of gametes were identified and characterized. Trinucleate cells of *T. brucei* with different DNA contents were observed; such cells can generate a mononucleate gamete and a binucleate cell with unequal DNA content via cytokinesis. From the binuclear cell, three more gametes are produced after two consecutive divisions. Thus, gamete formation in this trypanosomatid is considered a meiotic event with sequential production of haploid gametes. Despite the lack of experimental evidence, there is still the possibility that type 2K1N and 1K1N isogamous gametes play the role of male and female gametes (Peacock *et al.*, 2021). On the basis of the evidence available to date, it can be said that *T. brucei* undergoes a meiotic sexual reproduction phase with the formation of gametes and hybrids in invertebrate hosts.

## Sexual reproduction in *Trypanosoma cruzi*

Another trypanosomatid that incites the curiosity of researchers with regard to reproduction is *T. cruzi*, the causative agent of Chagas disease (American trypanosomiasis). Invertebrate hosts to *T. cruzi* include hematophagous insects of the family Triatominae, and vertebrate hosts include mammals, such as humans (Brener, 1973). The parasite can be transmitted to humans through the feces of triatomine insects, blood transfusion, laboratory accidents, ingestion of processed foods contaminated with parasites, and organ transplant, as well as congenitally, via the placenta (Casadei, 2010). *T. cruzi* has replicative forms, namely epimastigote in insects and amastigote in vertebrates, and infectious forms, metacyclic trypomastigote in insects and blood trypomastigote in vertebrates (Docampo et al., 2005; de Souza, 2009). 

Natural hybrids of *T. cruzi* (Sturm et al., 2003; Sturm and Campbell, 2010) and *in vitro* hybridization events between lineages have been previously reported, although, at the time, there was no evidence of the occurrence of meiosis (Brisse et al., 2003; Westenberger et al., 2005). However, recent studies on population genetics reported the possibility of sexual reproduction in natural populations of *T. cruzi* (Berry et al., 2019; Schwabl et al., 2019), which stimulates discussion about the reproduction process of this protozoan.

The first experimental evidence of genetic information exchange between *T. cruzi* individuals stemmed from *in vitro* experiments. Gaunt et al. (2003) analyzed two strains carrying different selection markers (hygromycin or neomycin). When mixed, the strains produced hybrid strains carrying both markers in amastigote forms within mammalian cells. The authors attributed such genetic exchange not to meiosis but to the fusion of diploid cells following chromosomal loss (Gaunt *et al.*, 2003; Weedall and Hall 2014). Evidence of *T. cruzi* hybrid formation was also reported in a recent study applying analysis of DNA exchange using thymidine analogs (ADexTA) (da Silva et al., 2018). The authors observed an increase in the fusion of epimastigote cells and genetic exchange events in naturally hybrid lineages (Alves et al., 2018), corroborating the findings of Gaunt *et al*. (2003) on the existence of genetic recombination in this parasite.

Although *T. cruzi* recombination processes involving gametic cells were not observed, the occurrence of genetic exchange in *in vitro* (Gaunt et al., 2003; Alves et al., 2018) and *in vivo* (Ramírez et al., 2012) populations, added with the evidence of meiotic sexual reproduction in natural populations (Messenger and Miles, 2015; Berry et al., 2019; Schwabl et al., 2019), suggest the possibility of a sexual phase with gamete formation. It is important to note that whereas direct observation of gametes both *in vivo* and *in vitro* confirms sex by meiosis, as in the case of *T. brucei*, non-observance of gametes is no definitive evidence that the species does not reproduce sexually (Cooper et al., 2007). Thus, it remains to be elucidated whether *T. cruzi*, in addition to performing genetic exchange, can undergo meiosis with gamete formation.

Parasexual recombination events involving trypanosomatids have been reported in *T. cruzi* (Gaunt et al., 2003; Schwabl et al., 2019) and in Leishmania (Sterkers et al., 2014), through genetic exchanges by nuclear fusion with reduced ploidy without the involvement of meiotic processes, as in fungi (Mishra et al., 2021). The parasexuality can be considered an alternative pathway to meiotic recombination, since during nuclear fusion recombination between parental genomes can also occur, increasing offspring diversity (Forche et al., 2008).

Among the trypanosomatids, it is known that the parasite *Leishmania sp* has a high degree of aneuploidy in its genome when compared to *T. brucei* and *T. cruzi* (Sterkers et al., 2011; Sterkers *et al.*, 2014; Shaik et al., 2021), which may hinder the occurrence of meiotic processes during recombination in this protozoan. Although Leishmania has meiosis genes in its genome, the increase in fusion events observed in this parasite when subjected to oxidative stress and the formation of polyploid hybrids (Louradour et al., 2022) support parasexuality events in this organism.

In contrast, *T. brucei* reproduces sexually by meiosis with stable ploidy (Peacock et al., 2011, 2014). Regarding the recombinational processes observed in trypanosomatids, it is possible that the parasite *T. cruzi* may be an intermediate form between Leishmania and *T. brucei*, presenting both parasexuality and meiosis mechanisms, depending on the environmental conditions. 

Alves et al. (2018) observed that epimastigotes of *T. cruzi* (CL Brener strain), modified to overexpress the RAD51 recombinase (from RecA protein family), showed a higher percentage of genetic exchanges when compared to wild-type cells. In the same work, it was also observed that the hybrids generated by the mixture between *T. cruzi* overexpressors of RAD51 were not diploid individuals equal to the parents, but parasites with altered ploidy and DNA content greater than that of the parents, typical of parasexual recombination events. However, wild-type and naturally hybrid cells of the CL Brener strain present in their diploid genome, two distinct haplotypes for each chromosome pair, which denotes the existence of an “ordered” regulation of genetic recombination mechanisms throughout evolution. This fact makes room for the occurrence of other recombinational events in *T. cruzi.*

## Meiotic proteins AND Recombinase DMC1

Meiotic proteins are found in the most diverse lineages of eukaryotes, constituting the so-called meiosis toolkit (Schurko and Logsdon, 2008; Hofstatter et al., 2020). These proteins have guided studies on the reproduction forms of various organisms, including protists. The major meiotic proteins are SPO11, which generates double-strand breaks to initiate meiotic recombination; HAP2, involved in gamete fusion processes; MHS4, MSH5, and MER3, associated with the resolution of crossing over; HOP1, responsible for aligning homologous chromosomes in prophase I; HOP2 and MND1, which assist in invasion and search for homology in meiotic recombination; REC8, which acts on the structural maintenance of chromosomes, responsible for the linkage between sister chromatids; ZIP1 and ZIP4, involved in synaptonemal complex formation; and DMC1, associated with recombination (Ramesh et al., 2005; Malik et al., 2008; Schurko and Logsdon, 2008; Hofstatter *et al.*, 2020). These proteins make up the meiosis toolkit and are fundamental to the maintenance of meiosis in all eukaryotes (Hofstatter *et al.*, 2020). Recombinase DMC1 (disrupted meiotic cDNA 1) is characteristic of meiotic events, as it is responsible for homologous recombination in meiosis.

Recombinase DMC1, first described in yeasts and found to play a central role in recombination events, synaptonemal complex formation, and cell cycle progression (Bishop et al., 1992), belongs to the meiosis-specific family RecA. It is responsible for genetic exchange between homologous chromosomes (Brown and Bishop, 2015). Individuals which reproduce sexually via meiosis contain recombinase DMC1 in their genome. However, there are two organisms that, despite reproducing sexually, do not have the DMC1 gene, probably as a result of gene loss events throughout evolution (Ramesh et al., 2005). One of these organisms is *Caenorhabditis elegans*. The nematode does not carry the *DMC1* gene but expresses RAD51 recombinase, which has similar characteristics and functions to meiotic recombinase. Fruit flies (*Drosophila melanogaster*) do not have some meiosis-specific genes but contain the gene encoding SPN-D recombinase, a protein with a similar role to DMC1 (Villeneuve and Hillers, 2001; Abdu *et al*., 2003). Of the protozoa addressed in this review, all carry the gene encoding DMC1, although experimental evidence of the importance of this gene has only been reported in *Plasmodium* and *T. brucei*. 

DMC1 knockout *Plasmodium* sp. showed problems in sporogonic development, with reduced oocyst numbers, and defective development of sporozoite forms in mosquitoes. These effects revealed the role of DMC1 in the sexual reproduction of this parasite (Mlambo et al., 2012). In *T. brucei*, the lack of DMC1 activity in repair by homologous recombination and antigenic variation (Proudfoot and McCulloch 2006), combined with high *DMC1* expression in sexual stages of the parasite, such as gametes and intermediates (Peacock et al., 2011, 2014, 2021; Howick et al., 2021), demonstrate the fundamental role of this recombinase in reproduction. These findings underscore the relevance of DMC1 or homologous proteins in meiotic recombination events and suggest that individuals who reproduce sexually contain functional recombinase DMC1 in their genomes. Nevertheless, it remains unknown whether the opposite applies, that is, if all individuals who have DMC1 are able to reproduce sexually.

Proteins of the RecA family, such as meiosis-specific recombinase DMC1, interact directly with single-stranded DNA during genetic recombination. These interactions occur in specific regions of the protein, known as DNA binding motifs or loop1 (L1) and loop2 (L2) regions (Chen et al., 2008). According to the literature, these regions are well preserved among eukaryotes that are known to undergo meiosis (Steinfeld et al., 2019). 

**Figure 1 -:**
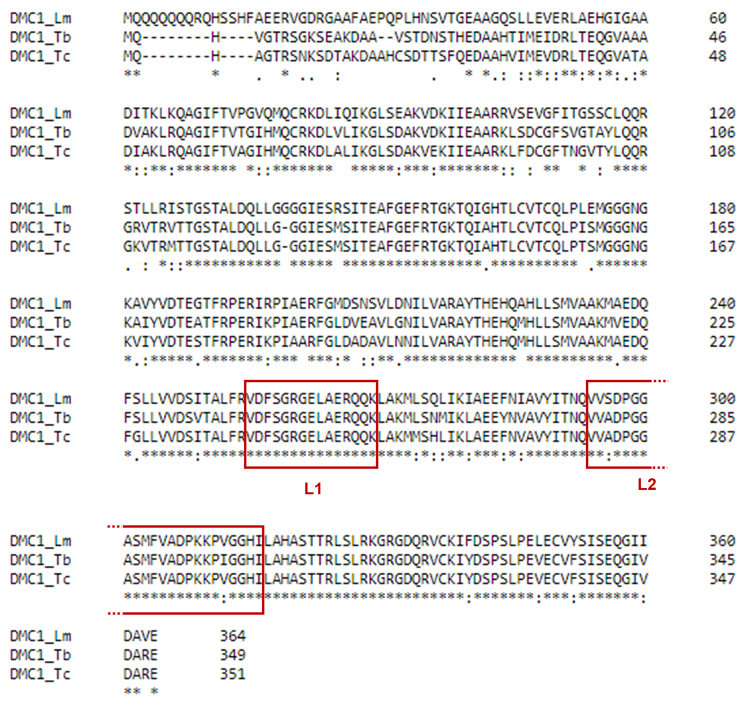
DMC1 sequence alignment between Leishmania and trypanosomes. Amino acid sequences of meiosis-specific recombinase DMC1 were obtained from the TriTrypDB database. DMC1_Lm, *Leishmania major* (ID: LmjF.35.4890); DMC1_Tb, *Trypanosoma brucei* (ID: Tb927.9.9620); DMC1_Tc, *Trypanosoma cruzi* (ID: TcCLB.506885.310). Loop1 and loop2 regions are highlighted by red boxes. Alignment was performed using the multiple sequence alignment function of Clustal Omega.

In analyzing the protein sequences of recombinase DMC1 in the trypanosomatids addressed in this review (Figure 1), we observed high conservation among amino acid sequences, especially in DNA interaction regions, such as L1 and L2. *Leishmania*, *T. brucei*, and *T. cruzi* shared 100% sequence identity in L1 and 91% in L2, demonstrating the conservation of DMC1 structure and function, essential to the meiotic machinery.

**Figure 2-:**
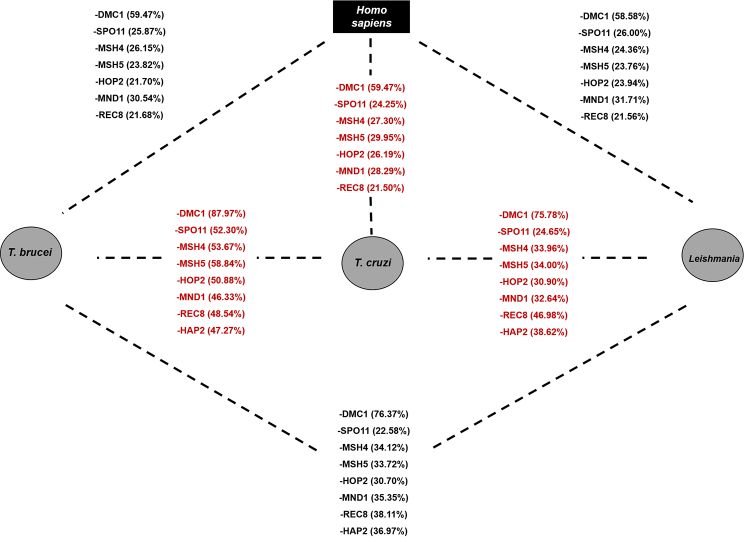
Sequence identity of meiotic proteins found in trypanosomatids. Comparison of meiosis toolkit proteins sequences between *Trypanosoma brucei*, *Trypanosoma cruzi*, *Leishmania major*, and *Homo sapiens*, for which these proteins are well characterized. Dashed lines represent comparisons between different organisms. Meiotic proteins of *T. cruzi* shared with *T. brucei*, *Leishmania*, and humans are highlighted in red. Trypanosomatid protein sequences used for comparisons were obtained from the TriTrypDB database and human protein sequences were acquired from GenBank, according to the accession numbers listed in Table S1. Sequence identity was assessed using Clustal Omega. Amino acid sequences were aligned individually and paired between organisms. The percentage of identical amino acids is shown in parentheses next to each protein analyzed.

In analyzing the sequences of other meiotic proteins, such as those that make up the meiosis toolkit (see Figure 2), we observed that almost all are annotated in the genome of trypanosomatids, except for ZIP1 and ZIP4, which are involved in the formation of the synaptonemal complex. This does not rule out the possibility, however, that other proteins play a similar role in trypanosomatids. Regarding HOP1, the HORMA domain was only annotated in the *T. brucei* genome (Tb927.10.5490), precluding comparison with other trypanosomatids. This comparative analysis between meiotic protein sequences suggested the possibility of sexual reproduction in all evaluated trypanosomatids and the existence of sexual phases with gamete formation, not yet observed in *T. cruzi* or *Leishmania*. Such findings may guide future studies on the occurrence of cryptic sex in these organisms.

In *T. cruzi*, to date, all hybrids were identified to have been generated through nuclear fusion between parental cells, without the presence of gametes or cells with haploid DNA content. However, the occurrence of meiotic allele segregation in *T. cruzi* populations (Schwabl et al., 2019) makes us wonder why gametes have not yet been observed *in vitro*. 

One of the explanations is anti-recombination, frequent between interspecific crosses, by which different species with divergent DNA sequences produce aneuploid and infertile hybrids (Kao et al., 2010; Gilchrist and Stelkens, 2019). For meiotic recombination to occur, pairing of homologous chromosomes must occur in meiotic prophase I, and, as is already known, the reduction of homology between DNA molecules can decrease recombination efficiency. Examples of reduced genetic exchange events between organisms that have some degree of heterology can be found in both prokaryotes (Rayssiguier et al., 1989) and eukaryotes (Hunter et al., 1996). *T. cruzi* hybrids have been generated in the presence of genetically modified cells carrying exogenous genes, such as antibiotic resistance genes. This fact might hinder the observance of possible gamete forms in this parasite. 

Anti-recombination is dependent on DNA mismatch repair (MMR). During meiosis, pairing of chromosomes with genetic divergences is hindered, and MMR proteins seem to play a key role in this process (Borts et al., 2000). Of note, MMR proteins, such as MSH4 and MSH5, involved in crossing over, are essential for meiosis to occur satisfactorily. In the absence of MSH2 and PMS1, which are involved in MMR in yeasts, hybrids show reduced polyploidy and increased viability of reproductive forms, thereby demonstrating the role of repair systems in the case of poor base pairing during meiosis in hybrids with heterologous DNA (Matic et al., 1995; Hunter et al., 1996; Gilchrist and Stelkens, 2019). In other words, in the presence of MMR, non-homologous DNA sequences fail to pair and therefore do not originate gametes with reduced haploidy.

As mentioned before, the *in vitro* study on *T. cruzi* hybrids revealed the occurrence of fusion events with genetic exchange in the naturally hybrid strain CL Brener as well as an increase in DNA content in hybrids generated from modified cells (Alves et al., 2018). If anti-recombination does indeed occur in genetically modified *T. cruzi* hybrids, the formation of polyploid parasites and non-observance of haploid gametes would be justified. One of the ways to test this hypothesis would be to analyze whether the expression of proteins involved in MMR is upregulated in *T. cruzi*. Another strategy would be to generate mutant cells for these proteins and observe the formation of hybrids.

As depicted in Figure 2, *T. cruzi*, whose form of sexual reproduction remains to be elucidated, shares several meiotic proteins with other trypanosomatids and humans. Meiotic proteins of *T. cruzi* and *T. brucei* share 50% sequence identity or more. Regarding DMC1, the parasite exhibits high sequence identity with *Leishmania* sp. (75%) and *T. brucei* (90%). The facts that *T. brucei* can reproduce sexually (with gamete formation by meiosis) and carries the *DMC1* gene as well as all genes participating in the meiotic machinery suggest the occurrence of sexual reproduction in *T. cruzi*, a parasite with high intraspecific genetic diversity (Zingales et al., 2009; Zingales *et al.*, 2012). Natural hybrids of *T. cruzi* are responsible for the majority of Chagas disease cases in countries of the Southern Cone (Miles et al., 2009), and meiosis, although not yet observed *in vitro*, might be contributing significantly to this genetic variability found in nature.

## Conclusion

The number of individuals of the protist kingdom that can reproduce sexually may be much higher than previously believed, given that many have cryptic sex, as seems to be the case of *Leishmania* and *T. cruzi*, and many others have not yet been studied. Here, we cited examples of different parasites capable of recombining their genetic material through meiotic sex with gamete formation. These organisms have developed the most varied strategies for species perpetuation throughout evolution. Interaction of these protozoa with their respective hosts shaped the form of disease transmission, for which genetic variability provided by sexual reproduction was a determinant factor.

In Apicomplexa parasites, addressed in this review, obligate sexual reproduction with the presence of haploid gametes is fundamental for parasite transmission, as observed in *Plasmodium* and *Toxoplasma*. Such a characteristic may be targeted by epidemiological strategies to contain the spread of malaria and toxoplasmosis (Cruz‐Bustos et al., 2021). Understanding if and how meiotic recombination events occur in trypanosomatids such as *T. cruzi* and *Leishmania* can guide the development of different research methods for these organisms. Furthermore, this information may contribute to the acquisition of new knowledge in both basic and applied science for the control of neglected diseases.

## Supplementary material

The following online material is available for this article:

Table S1 - Accession numbers of the meiotic protein sequences analyzed.
