# Supplementary material for: Sex in protists: A new perspective on the reproduction mechanisms of trypanosomatids
Source: Genet Mol Biol. 2022 Oct 10;45(3):e20220065. doi: 10.1590/1678-4685-GMB-2022-0065 (PMC9552303; doi:10.1590/1678-4685-GMB-2022-0065)
Supplement: Table S1 - [file 1415-4757-GMB-45-3-e20220065-s1.pdf]

Genetics and Molecular Biology
https://doi.org/10.1590/1678-4685-GMB-2022-0065

Supplementary Material to "Sex in protists: A new perspective on the reproduction mechanisms of trypanosomatids"

Table S1. Accession numbers of the meiotic protein sequences analyzed. *Trypanosoma cruzi*, *Trypanosoma brucei*, and *Leishmania* sequences were obtained from the TriTrypDB database and human sequences from NCBI.

Gene	Accession number			
	T. cruzi	***T. brucei***	***Leishmania***	***Homo sapiens***
DMC1	TcCLB.506885.310	Tb927.9.9620	LmjF.35.4890	CAG30372.1
SPO11	TcCLB.503619.10	Tb927.5.3760	LmjF.36.2780	AAD52562.1
MSH4	TcCLB.509967.20	Tb927.10.1270	LmjF.21.0885	AAB72039.1
MSH5	TcCLB.506237.10	Tb927.3.4280	LmjF.29.1710	BAB63375.1
HOP2	TcCLB.511627.130	Tb927.2.5190	LmjF.27.2420	NP_057640.1
MND1	TcCLB.508647.10	Tb927.11.5670	LmjF.24.1010	NP_115493.1
REC8	TcCLB.508387.170	Tb927.7.6900	LmjF.05.1090	NP_001041670.1
HAP2	TcCLB.509105.4	Tb927.10.10770	LmjF.35.0460	
